# Prevention of road crashes in older adults: perspectives on facilitators, barriers and the role of the family doctor

**DOI:** 10.1186/s12877-021-02569-0

**Published:** 2021-11-06

**Authors:** Mario Rivera-Izquierdo, Luz María Valverde-Cano, Virginia Martínez-Ruiz, María Rosa Sánchez-Pérez, Francisco Javier Atienza-Martín, Luis Miguel Martín-delosReyes, Eladio Jiménez-Mejías

**Affiliations:** 1 Department of Preventive Medicine and Public Health, School of Medicine, University of Granada, Avenida de la Investigación 11, Edificio A, 8ª planta, 18016 Granada, Spain; 2 Doctorate Program in Clinical Medicine and Public Health, University of Granada, Granada, Spain; 3 Instituto de Investigación Biosanitaria de Granada (ibs.GRANADA), Granada, Spain; 4 Chair of Teaching and Research in Family Medicine SEMERGEN-UGR, University of Granada, 18016 Granada, Spain; 5 Centros de Investigación Biomédica en Red de Epidemiología y Salud Pública (CIBERESP), Granaha, Spain

**Keywords:** Road safety, Older drivers, Family doctor, Focus group, Qualitative research

## Abstract

**Background:**

People over 64 years have a high fatality rate when they are involved in traffic accidents. Besides, older victims of road crashes are expected to rise in the future due to population aging. The purpose of the study was to document their perception on the role of the family doctor, the main facilitating factors, and the perceived barriers to the temporary or permanent restriction of their driving.

**Methods:**

This qualitative study used focus group methodology. A sample of 16 people over 65 years old was obtained through a series of segmentation criteria at an active participation centre for older adults in a small town in Jaén province (Spain). All were invited to participate in a discussion during which they were asked to express their opinions and subjective experiences concerning the role of their family doctor. The group conversation was taped, fully transcribed and analysed, and codes were generated with both deductive and inductive methods.

**Results:**

After merging the codes to generate themes, we identified 9 relevant categories: perception of age-related risk, road safety, role of public authorities, driver assessment centre, role of the family doctor, role of the family, proposals for addressing traffic accidents in older adults, consequences of the driving prohibition, and public transport. All categories help to explain the subjective driving and traffic safety experiences of older road users.

**Conclusions:**

Although family doctors do not usually ask their older patients about road driving, they are highly valued by these patients. Thus, family doctors have a great potential to act, along with the family members, for the benefit of older patients’ traffic safety, in ways that can prevent their involvement in road crashes and reduce the negative consequences of having to stop driving if necessary.

## Background

Populations in developed countries are aging, and the average age of drivers is concurrently rising [1–3]. In Spain, older adults represent 19 % of the general population and 15 % of the drivers’ census, which is an official register maintained by the Spanish General Traffic Directorate including information on every person holding a driving license in Spain: age, sex, type of license and date of license. According to the Spanish General Traffic Directorate [3], 102,299 traffic accidents with victims were reported in this country in 2018. Most of the fatalities in these road crashes were in people between 35 and 44 years old; however, considering the death rate per million population, the age group with the highest rate was 75 to 84 years (64 deaths per million population), followed by the age group over 84 years (61 deaths per million). Specifically, people over 64 years old were involved in 11,647 road crashes, and 496 people of this age group died (66 % of whom were male). Also, the 3.8 fatality rate for this age group was more than 3-fold the rate in the rest of the population of victims of traffic accidents, and this rate increased with age [3]. These numbers are expected to rise in the future if current trends hold. Thus, the steady increase in the over-65 driver population poses a relevant public health issue.

Age-related diseases, associated medications, and overall physiological age-related decline have been reported to affect the skills needed for safe driving and may even predispose pedestrians to involvement in an accident. These skills mainly involve vision, hearing, and cognitive, physical, and psychomotor status [4]. In fact, once a certain level of psychomotor deficit has been reached, compensatory behaviours such as speed reduction are not enough to prevent traffic accidents [5]. Also, some medications frequently used by people over 65 years old can compromise driving skills, e.g., hypoglycaemic agents, antihypertensives, antidepressants, and sedative-hypnotics [6]. Furthermore, because of their vulnerability and fragility, the consequences of involvement in a road crash are more severe in older adults than younger people [4]. Information on the above factors, as potential contributors to the likelihood of involvement in traffic accidents in older drivers, is relevant for the development of preventive programs [7].

Given that people over 65 years of age are the population group that most frequently visits health centres, family doctors can do important work in preventing road crashes among these users. In Spain, family doctors (also known as family physicians or general practitioners) complete a 4-year specialisation after their undergraduate training. They mainly perform their work in the primary healthcare centres or in the emergency services, providing comprehensive and continuous care to every individual who requests medical assistance. Furthermore, they are concerned not only with the individual patient, but also with the patient in the context of the family and with the family in the context of the community [8]. Their proximity and the longitudinal nature of their care have contributed to placing them among the highest valued healthcare professionals in Spain [9].

Thus far, the role of family physicians in crash prevention area has not been well defined, nor is raising patients’ awareness among their main objectives. Nevertheless, a few studies have proposed some standards and have been useful in preparing action guidelines [10–12]. What seems obvious is that family doctors have considerable influence on their patients. Therefore, their intervention in this public health issue is potentially a key factor in improving road safety in older adults. For example, doctors could give advice about driving skills, or about whether the patient should stop driving temporarily or permanently if they are not fit for this task. However, for many patients, loss of their driving license may have a major impact on their quality of life, leading to feelings of depression, isolation, and loss of freedom and autonomy [13]. If driving is considered temporarily unsafe, or if the patient should permanently cease driving, it is essential to discuss alternative transportation options by (for example) asking if they have close relatives or friends willing to assist them with transportation, and if not, finding ways to use public transport [14].

Despite the relevance of this topic, few qualitative studies have investigated the role of family doctors in older adults’ road safety, and specifically in Spain, no such research has been carried out to date. In the present qualitative study, we aimed to find out what health centre users over 65 years old think about the involvement of their physician in reaching a decision on their driving aptitude. We wished to know what drivers in this vulnerable group actually experience in connection with their driving, i.e., whether they are aware of the risks depending on their individual characteristics, whether they would accept a recommendation to use alternative transport (public, family, or friends), whether they would feel their autonomy was reduced, and what other concerns they may have. This information would make it possible to take appropriate measures within a particular contextual framework rather than simply following guidelines, in which patients’ opinions have no place.

Therefore, the aims of this qualitative study were threefold: (1) to explore older road users’ previous experiences regarding their perception of the risks associated with their age, medication use, and their clinical condition; (2) to document their perception on the role of the family doctor, the main facilitating factors, and the perceived barriers to the temporary or permanent restriction of their driving; and (3) to shed light on how the social and family environment influences older adults’ mobility patterns and the decision to cease driving if necessary.

## Methods

### Study design

This research was designed from a qualitative methodological perspective. The methodological approach and thematic content analysis used here are included within the phenomenological paradigm, as well as in grounded theory [15, 16].

### Setting

The town of Alcalá la Real (population 21,605) in Jaén province, southern Spain, between March and April 2018. Approximately two thirds of the population of Jaén live in rural settings, mainly in small villages. This means that people have to travel to larger population centres most of the time (e.g., to go to hospital or to obtain certain goods and services). The public transport (mainly bus) is managed by the Jaén Transport Consortium [17]. Although fairly well organized, the frequency of vehicles on some routes is not very high, leading many citizens to choose private vehicles to avoid depending on timetables.

### Study population

People over 65 years old who visited the Active Participation Centre for Older Adults in Alcalá la Real. The inclusion criteria were: (1) age over 65 years, and (2) road user as a pedestrian or motor vehicle driver. The exclusion criteria were: (1) cognitive impairment or psychiatric or psychological disorders that might have interfered with the ability to understand, participate in, or express opinions on the issues raised during the discussion, and (2) not providing voluntary informed consent both verbally and in writing.

### Sample

Intentional qualitative sampling was used to select participants based on a series of segmentation criteria: (1) gender (male, female), (2) type of road user (pedestrian, motor vehicle driver, passenger, public transport user), (3) presence and severity of pathologies that *a priori* may pose a higher risk of traffic accidents (vision or hearing impairment, obesity, diabetes, heart disease, obstructive sleep apnoea syndrome, Parkinson disease, etc.), and (4) use of medications which may compromise road safety (opioid analgesics, benzodiazepines or other anxiolytics, hypotensive drugs, oral antidiabetics or insulin, etc.) (see below). These criteria were then used to generate different profiles. The choice of these segmentation criteria was informed by a previous theoretical approach based on the judgment of a panel of experts and through direct dialogue with staff at the Active Participation Centre for Older Adults, and with a key worker from this centre who acted as a gatekeeper, providing access to the participants. In addition, we used snowball sampling as a complementary method.

A total of 16 participants among users of the centre were included in the final sample. Their main characteristics, which were self-reported, are shown in Table 1.

**Table 1:** Characteristics of the sample of older adults who provided experiences and opinions on driving

No.	Gender^a^	Age	Civil status	Visits to their physician	Type of road user	Pathologies	Medications^b^
1	M	80	Single (with stable partner)	1 per year	Driver and pedestrian	Hypertension, heart failure, herniated discs, difficulty walking	Enalapril, bisoprolol, furosemide, tramadol, simvastatin, acenocoumarin, others
2	F	74	Married	3 per year	Pedestrian and nearside passenger	Difficulty walking (hip surgery), coagulation disorders	Paracetamol, metamizole, tramadol, diazepam, acenocoumarin
3	M	77	Married	1 per 2-3 months	Driver and pedestrian	Parkinson disease, hypertension, transmission hearing loss	Captopril, drugs for Parkinson disease
4	M	78	Married	1 per 2 months	Driver and pedestrian (mostly)	Hypertension, diabetes, essential tremor	Metformin, losartan, furosemide, omeprazole, metoclopramide, others
5	M	67	Single	1 per several years	Driver and pedestrian	None	None
6	F	69	Divorced	1 per month	Pedestrian	Asthma, hypertension, diabetes	Salbutamol, budesonide, metformin, insulin, omeprazole, paracetamol, simvastatin, enalapril
7	M	87	Widower	1 per year	Accompanied driver and pedestrian	Diabetes, vertigo, hearing loss, difficulty walking, OSAS, obesity	Insulin, sulpiride, betahistine, omeprazole, oxygen mask, others
8	F	69	Married	4 per year	Driver and pedestrian	Unilateral amaurosis, bipolar disorder, osteoporosis, osteoarthritis	Lithium, benzodiazepines, chondroitin sulfate, alendronate, vitamin D, metamizole
9	F	80	Widow	1 per 2 months	Pedestrian	Thrombosis, hearing loss, depression, hypothyroidism, osteoarthritis, others	Acenocoumarin, sertraline, diazepam, levothyroxine, vitamin d, paracetamol, ibuprofen, diclofenac, omeprazole
10	M	82	Married	2 per year	Pedestrian and driver (only to go to the nearest town)	Hypertension, benign prostatic hyperplasia, presbyopia, presbycusis, osteoporosis, cataracts	Tamsulosin, acenocoumarin, antihypertensives, others
11	M	79	Married	Medical check-ups	Driver and pedestrian	Hypertension, osteoarthritis, difficulty walking, COPD	Ibuprofen, medication for hypertension and COPD
12	M	80	Married	1 per 1-2 months	Driver and pedestrian	Benign prostatic hyperplasia, hypertension, depression, atrial fibrillation, digestive tract ulcer	Tamsulosin, enalapril, diazepam, sertraline, omeprazole, ranitidine, acenocoumarin, others
13	F	69	Married	3 per year	Pedestrian	Allergy, hypothyroidism, herniated discs, hypercholesterolemia	Simvastatin, L-thyroxine, antihistamines, metamizole
14	M	71	Divorced	1 per several years	Driver (day and night)	Benign paroxysmal vertigo, hypercholesterolemia, others	Betahistine, omeprazole, atorvastatin, others
15	M	67	Married	Medical check-ups	Driver and pedestrian	Diabetes, hypertension	Drugs for diabetes and hypertension
16	M	80	Married	1 per 1-2 months	Driver and pedestrian	COPD, hypertension, allergy, atrial fibrillation, osteoporosis	Azithromycin, ipratropium, furosemide, apixaban, enalapril, beta-blockers, acetylcysteine, anticoagulants and antiaggregants

OSAS, obstructive sleep apnea syndrome; COPD, chronic obstructive pulmonary disease

^a^ Male (M), Female (F)

^b^ Medications not specified by the participants were recorded as “other” or “drugs for a specific disease”

### Data collection and procedures

We conducted a focus group with all sixteen participants. A focus group is considered a variant of a discussion group, in which participants may be a pre-existing group of people, as in this case (users of centre who knew each other previously) [18]. This approach has been widely used in the field of health research, e.g., in health education and health promotion. According to Morgan [19], interactions among focus group members produce data and insights that would be less accessible without such face-to-face interaction.

**Fig. 1:**
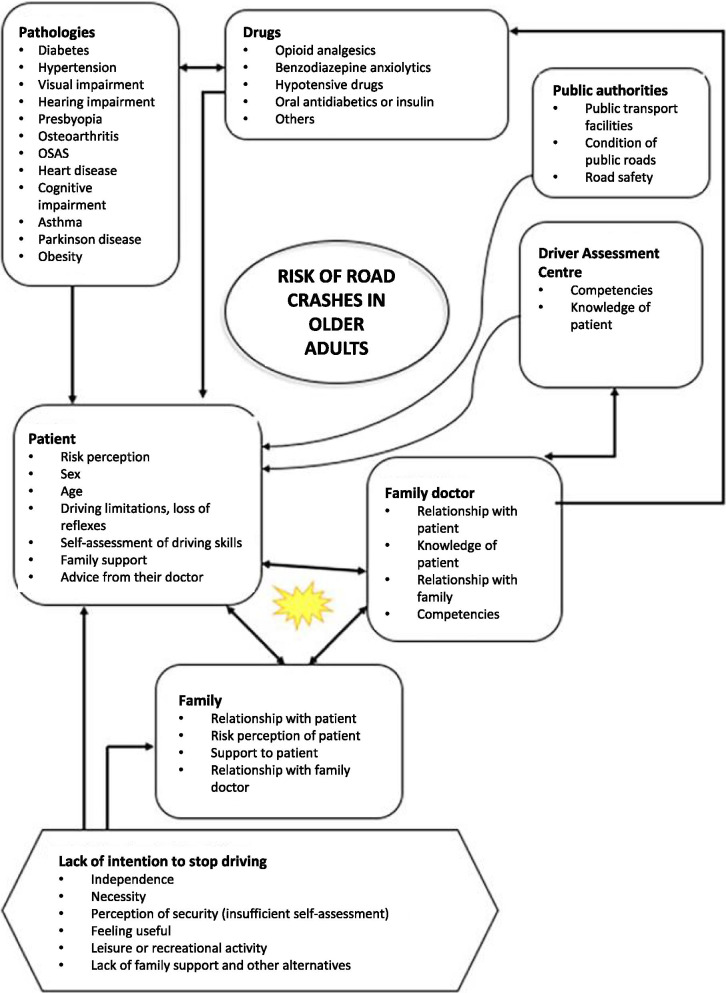
Map of sampling units, ideas, topics to discuss and potential relationships between them. *The core of the debate centers on these three ideas: patient, family doctor, and family

An inductive approach was used to identify a series of themes and categories that could influence respondents’ subjective opinion on the role of the family doctor in preventing traffic accident-related injuries in older adults. To this end, the research team conducted an extensive literature review, which was combined with multiple meetings to discuss what these factors would be and how they would relate to each other. The resulting topics, along with information related to the sampling units and possible relationships, are shown in Fig. 1. In light of the topics identified in this step, a set of thematic research questions was developed, which we later transformed into a semi-structured discussion guide that the main interviewer used in the focus group. The initial question posed to participants was *In recent years, traffic mortality has been increasing mostly in people over 65 years old. What are your thoughts about this?* Table 2 presents the questions contained in the discussion guide.

**Table 2:** Research questions covered in the discussion guide

Research questions	Interview questions
*How serious is the problem perceived to be?*	Do you think traffic accidents are frequent in older adults? Does the topic of car accidents in older adults seem an important issue to you? Do you perceive road use as dangerous because of traffic accidents involving older people?
*What are the participants’ mobility patterns?*	How do you usually get around to go places: walking, driving a vehicle, as passengers, public transport, other? How many of you are pedestrians and how many are drivers?
*What are the main barriers to stopping driving temporarily or permanently?*	How would you feel if for some reason you were advised to stop driving, temporarily or permanently? What problems would you perceive if you were prohibited from driving? How would you feel if your driving license was revoked?
*What are the main reasons to continue driving despite knowing that they belong to a group at high risk of accidents?*	Are you considering using other alternatives instead of driving? If not, why? If you perceive that driving is increasingly difficult as you get older, why don’t you avoid driving?
*According to their experiences, what role do participants subjectively attribute to the family doctor?*	Do you think that your family doctor could have a role in the matter we are discussing? What role should your family doctor have in this matter? Could your family doctor influence your decision to drive or not?

 The group discussion took place in a meeting room at the active participation centre and lasted 1.5 h. All participants were informed about the purpose of the meeting. Anonymity and confidentiality of their responses were assured, and their permission to tape the discussion was obtained. The moderator posed questions to the whole group and redirected the discussion, when necessary, in a neutral manner. A second researcher acted as an observer, documenting participants’ nonverbal language and other contextual details (e.g., silences, facial expressions, interruptions, ambient noise, etc.).

 The group conversation was fully transcribed, and then shown to all participants for their review and approval. All participants stated that their transcribed contributions to the discussion were complete and accurate.

### Analysis

The transcripts of the different observations constituted the primary documents of the hermeneutical unit. All transcripts were read closely to identify emerging themes, and deductive coding was used with the components described by Verkade at al. [20] as codes (code by list). Therefore, inductive and deductive approaches were mixed to enrich the research, as frequently performed in qualitative research [21, 22]. Additionally, as live codes, (code in vivo), components that emerged outside the proposed list were added. Subsequently, the coded fragments were refined and modified if necessary, and initial codes were reconfigured, if necessary, prior to their grouping into categories. Uncoded material was reviewed to detect additional important themes in participants’ conversation, which were also coded freely. The categories and codes finally selected are the product of the consensus (triangulation) of three of the researchers who independently analysed the transcripts.

The texts were imported into NVivo 11 qualitative data analysis software for the coding process.

### Ethics

 The management, notification and transfer of personal data for all participants met all the requirements set forth in Spain’s Organic Law 3/2018, of December 5 on the protection of personal data and guarantee of digital rights and complied with the requirements of Law 14/2007 (Biomedical Research) regarding the protection of personal data and the guarantee of confidentiality.

 All participants were given an information sheet that explained the objectives and activities of the study in detail, and were also given documentation regarding informed consent, which explained the voluntary and not-for-profit nature of participation in the study. By law, these documents must be signed by each participant and kept on file by the principal investigator of the study. Only those persons who freely and voluntarily agreed to participate in the study and provided their informed consent in writing were included in the study.

Permission to approach users of the centre was requested from, and granted by, the management of the Active Participation Centre for Older Adults in Alcalá la Real.

## Results

A total of 16 participants aged 67 to 87 years took part in the focus group (69 % males). After merging the codes to generate themes, we identified 9 relevant categories from the narratives, which are shown in Table 3 along with the corresponding subcategories. Saturation level was reached. Below we cite the most indicative quotes drawn from the thematic content related to each category.

**Table 3:** Categories and subcategories

Categories	Subcategories
*Perception of age-related risk (yes/no)*	Yes: Attitude or behaviour changes or decision-making because of risk perception No perception of age-related personally. Instead: ­ Consideration of the rest of society as a threat ­ Changes in roads, driving rules, requirements, etc. ­ Habit of driving frequently ­ Positive driving experience ­ Assumption that driving is a risk in itself, not something that can be prevented ­ Denying age-related physiological changes in oneself Self-assessment of driving skills
*Road safety*	Traffic violations Road user education
*Role of public authorities*	Complaints regarding infrastructure Perception of public institutions as an enemy Duties of public institutions
*Driver examination centre*	Perception of importance License renewal as a justification for maintaining one’s driving skills Perception of incompetence (conflict: knowing of someone whose license was renewed, but was not fit to drive) Response to denied license renewal
*Role of family doctor*	Opinion on who knows the driver best (family doctor vs. driver examination centre doctor) Family doctor role (in practice) in their patients’ driving Opinion on whether the doctor should have authority to influence driving Importance of the family doctor
*Role of family*	First to perceive the risks for driving Communication with the family doctor Actions on a family member’s driving Support for a family member who should not continue driving
*Proposals to address the problem (traffic accidents in older adults)*	Family doctor’s role Driver examination centre’s role Family roles Self-evaluation
*Consequences of the prohibition to continue driving*	Feelings Acceptance Denial (despite prohibition or recommendation to stop driving) Alternatives if driving is stopped
*Public transport*	Perception of frequent use as a facilitator in the use of public transport Perception of need for use Disadvantages, obstacles, difficulties with use Car as a personal convenience

### Perception of age-related risk

Most participants recognized that driving-related risks increase with age, mainly because of the increasing frequency of physical and mental deterioration. Some of them described compensatory behaviours to try to reduce this risk, such as not driving at night, or even giving up use of their vehicle to avoid “temptation”.

*“The problem in old drivers is that your feet don’t obey you, and when you go to step on one thing, you stepped on the wrong thing”* (Participant 4).

*“What we have to do is be careful because we don’t have those reflexes anymore, and you can’t see a car off in the distance”* (Participant 8).

*“I had a friend … who was 86 and still allowed to drive for two more years, but he said, ‘I’m selling the car; that way I won’t have any problems because if it’s parked right outside my door, I’ll use it’. When he wants to go out to the farmhouse, we give him a ride, and that’s that. But he was in good shape even though he said he wasn’t able to drive”* (Participant 1).

However, although participants were generally aware of age-related risks, many of them did not believe that this risk applied to them in particular. Instead, they shifted blame to the rest of society (*“We older folks go slower and everyone else goes really fast and they ignore the rules”*), to the changes in their driving environment (*“You use this street your whole life with no problem, and now, all of a sudden, you turn into it out of habit because you didn’t see the sign”*), or to the assumption that traffic accidents are inevitable (*“As long as there are cars there will be accidents; that’s the way things are”*) (Participant 3).

### Road safety

Almost unanimously, participants complained about noncompliance with traffic regulations by some road users and highlighted the important role of road user education to avoid accidents.

*“But there are very few deaths considering how often the traffic signs are ignored”* (Participant 6).

*“Road user education: the pedestrian should know when to use a pedestrian crossing, and pedestrians have right of way, relatively speaking. If a car is approaching fast you don’t have right of way; don’t start crossing and wait”* (Participant 10).

### Role of public authorities

Some participants, especially drivers, expressed their dissatisfaction with certain urban elements that could limit their vision, such as containers or *“gardens […] you see the pedestrian when he is already on the road”.* Sometimes they perceive public institutions as an enemy that imposes fines on them even if they have not committed any infraction (*“I got fined even though I was within the speed limit”*) (Participant 12).

Some of them called for a greater involvement of public institutions to facilitate compliance with traffic regulations by older adults.

*“The traffic code is falling behind for older people because some things are not really up to date…”* (Participant 14).

*“The traffic rules need to be updated for older drivers; the road signs have changed”* (Participant 16).

### Driver assessment centre

In Spain, class B (passenger cars) driving licenses are valid for 10 years (up to 65 years old). For drivers over 65 years old, the validity period is 5 years. To renew the license, it is necessary to pass a medical examination to prove that the user has an adequate psychophysical state for driving. This test is carried out in privately managed driver test centres. In most of these centres it is not mandatory to present any document from the family doctor providing information on health problems or prescribed medication. The collection of these data is done verbally. Full credibility is given to the information of users. This situation makes it possible that drivers do not always remember all their health problems, or that they conceal the most reprehensible aspects for safe driving.

Participants had a favourable perception of their local driver examination centre, particularly of the doctors who work there. In this connection, it emerged that part of their satisfaction was because they considered renewal of their driving license as proof that they maintained good driving skills.

*“My doctor here said no, and I went to Jaén* [capital city of the province]; *the doctor there is more important and has seen that I’m fine, and now I have a two-year license renewal”* (Participant 1).

Nevertheless, participants also expressed their concern about the centre not always fulfilling its duty to carry out thorough medical examinations, preferring instead to prioritize the income from application fee payments. On this point, some of them defended the role of their family doctor as the person who has in-depth knowledge of their clinical status (*“I say the family doctor is the one who knows you better, because the traffic exam centre doctor doesn’t know who you are”*) (Participant 15). Some participants mentioned friends of theirs whose license was renewed even though they were not fit to drive.

*“When you go for your renewal physical and take all the tests, and give them the 40 euros, then you can drive. What the hell?! That’s what I see”* (Participant 14).

*“I went to renew my driver’s license, and they didn’t give me any tests at all. I just paid the fee. I was surprised because that way, anyone can go* [apply for renewal], *from the looks of it”* (Participant 8).

*“I know an older person who has a driver’s license, but that person wasn’t fit any more because of tremors”* (Participant 9).

Also, many participants recognized that if the examination centre did not issue a favourable report in support of their license renewal application, they would try another centre.

### Role of family doctor

In general, participants had excellent opinions about their family doctor. They emphasized the importance of these physicians throughout the individual’s life, and as noted above, they believed their family doctor to have better knowledge of their clinical condition than the doctor at the driver examination centre.

*“We all need to see the doctor often, sooner or later”* (Participant 3).

*“The family doctor knows you better than the doctor at the driver examination centre because they know you have Parkinson’s or some other disorder in your feet, or if your knees hurt”* (Participant 7).

However, participants noted that their family doctor is not usually involved in decisions about their driving (*“Mine never says anything at all about driving”*) (Participant 11). When participants were asked if their physician should be authorized to participate in this issue, opinions were divided: some answered yes, some thought doctors should never get involved, and others opined these doctors should have an exclusively advisory role.

*“My opinion is that the official at the exam centre requests information from the family doctor, who knows the applicant better, in case they have any disease or anything”* (Participant 13).

*“I feel that the doctor should not play a role in this because the applicant is the person who knows best how they are, and after that comes the result of the physical exam at the license renewal centre”* (Participant 14).

*“Doctors know about the illnesses people have. It’s not their place to say, ‘Don’t drive’. Who is the doctor to say anything, although they should warn the patient”* (Participant 2).

### Role of family

Family members (especially spouses) were considered the first people to perceive the risks of driving in an older relative. One of the participants said, *“If I have any problem with driving, the first to know is my wife, my life partner”* (Participant 3). In addition, children and spouses usually communicate with the family doctor, who can let them know that their relative should not drive. Family members also try to convince older drivers to cease driving when they are no longer fit for the task – although they do not always succeed. Moreover, they are usually an important source of support, offering to drive their relative whenever needed.

*“His children told him no, but he wouldn’t stand them telling him no. He went off to the farmhouse, they worried about him, and then what happened, happened”* (Participant 7).

*“If I see I’m not well enough to drive, I resort to my granddaughter, my sister, or my daughter-in-law, and they all help”* (Participant 4).

### Proposals to address the problem of traffic accidents in older adults

According to our participants, involvement of the family doctor, driver examination centre, and family members is necessary to prevent traffic accidents in older adults, as noted above. They also highlighted the importance of being aware of their own limits as drivers.

*“The family doctor is the person who should see the family and tell them ‘Look, he’s (or she’s) not well enough to drive’”* (Participant 9).

*“They should take a test* [at the driver examination centre]*”* (Participant 10).

*“The doctor and family members should tell the person that they aren’t fit for that, and family members can just say ‘we’ll drive you anywhere and back home’”* (Participant 8).

*“When the time comes and I’m unfit to drive, I’m the one who’ll decide whether to drive or not because it depends on what’s going on in my life”* (Participant 15).

### Consequences of the prohibition to continue driving

Participants stated that being deprived of their driving license would affect their mood negatively (*“That’s the final blow. You’re tied hand and foot”*) (Participant 14). Although in general they would be willing to accept a driving ban, they also mentioned acquaintances who refused to comply with the recommendation or ban. Reasons for doing so were usually fear of losing their freedom and not wanting to inconvenience other family members.

*“When there’s a real justification, it’s to your benefit* [to stop driving] *because you might get into really serious trouble”* (Participant 16).

*“Now that the family doctor says he’s unfit to drive, what does he do? He asks to be transferred to a different doctor”* (Participant 2).

*“You like it because you can get around freely, and you don’t want to bother your family for rides, so you keep on driving”* (Participant 16).

If they had to stop driving, they believed they would have the support of their relatives or would use public transport (see below).

### Public transport

Participants perceived the advantages of public transport, such as its price and frequency, and considered it a likely alternative if they could not drive. However, they also noted some barriers to public transport, such as the inconvenience or the high cost of some modes of transport.

*“Sure, with local and intercity buses you have no problems or responsibilities, and with the discount card it’s cheaper”* (Participant 2).

*“Look, there’s a problem with the whole streetcar and city bus thing and all that. Now you have to go to a ticket office to buy a ticket, at that place, all full of people, and you’re about to miss your bus…”* (Participant 7).

*“Well of course, if you’re getting retirement benefits you might just as well hire a taxi”* (Participant 5).

Nevertheless, private vehicle use was perceived as the most convenient option, and the participants recognized that this was the main reason why they did not want to stop driving.

*“We prefer convenience and want to go wherever we want by car”* (Participant 3).

## Discussion

The main hypothesis of this study was that older age negatively affects collective road safety, and that reinforcing the role of the family doctor holds the potential to mitigate these negative effects.

The development of age-related diseases, the medications used to treat them, and physiological decline are some of the reasons why older age affects the skills required for safe driving – a proposition supported by numerous studies [4, 6, 14, 23, 24]. In Spain, deaths among persons 65 years old and over in accidents on interurban roads during 2018 increased by 31 % compared to 2017. Among different age subgroups, the number of deaths in road users 65 to 74 years old increased by 26 %, and in the group between 75 and 84 years old, deaths increased by 34 % in comparison to the previous year. In this same population group, accidents on urban roads during 2018 increased by 9 % in the 65- to 74-year-old group, and by 3 % in the 75-to-84-year-old group, with the only decrease being recorded in the group older than 84 years [3]. Greater involvement of the family doctor may be, at least in part, a potential solution to this public health issue. In favour of this proposition are the physicians’ knowledge about their patients (e.g., their overall health status and changes in their organic and psychological functioning), and knowledge about their patients’ pathologies and social environment (mainly, their family). Their frank, trust-based communication with patients and familiarity arising from relatively frequent contacts with older patients as compared to younger ones, along with the possibility of communicating with their family, are also relevant in this connection.

In practice, however, family doctors do not usually ask patients about their driving [25], and this was consistent with the views and experiences shared by our participants. The present findings show that patients’ opinions regarding the role that the family doctor could play in preventing traffic accidents and morbidity in older road users are neither uniform nor well defined. This theme occurred infrequently in the participants’ conversation and depended fundamentally on the personality of the doctor. Older patients continue to see this figure as a resource to consult for other health-related matters. In contrast, participants tended to attribute authority to judge the applicant’s fitness to the doctor at the driver examination centre, despite the scepticism most participants expressed regarding the reliability of this doctor’s judgement. Some explanations for these attitudes can be found in previous studies. For example, one study found that physicians usually face barriers to preventing traffic accidents among older people, such as the lack of validated tools to identify drivers at the highest risk of involvement in accidents, and the absence of guidelines on road safety for primary care physicians [26]. Another potential obstacle is that published studies on this topic are not widely disseminated among family doctors [27]. Furthermore, the scarce time that doctors can devote to each patient (the average duration of family doctor consultations in public health services is about 3 to 5 min), and the high volume of patients with a variety of medical problems, create stress for family doctors, which eventually curtails potential opportunities for preventive counselling [28].

In addition, family physicians must face their patients’ own difficulties with self-evaluation. Generally, although older patients recognize their physical and cognitive decline, they do not relate it to the possibility of causing traffic accidents. In most cases, they believe that they can overcome these deficits with compensatory measures, such as not driving at night or at times when traffic is heaviest [24]. Moreover, these patients frequently reject alternatives to driving, e.g., the use of public transport or dependence on family members [29]. According to our results, cessation of driving is a sensitive topic that generates anxiety in older adults. Among other factors, their concerns arise from their feelings of uselessness and loss of independence, freedom, autonomy and health [30]. For this reason, family doctors may fear that the suggestion to stop driving may deteriorate the doctor–patient relationship [31].

Nevertheless, family doctors also face a number of opportunities to address this problem directly or indirectly. Despite difficulties with their self-evaluation and self-perception of risk, older adults recognize their limitations, and are reasonable about accepting their doctor’s advice [24]. For their part, physicians are generally willing to provide advice on road safety if they are trained in how they should do so, in light of their ethical and legal obligations regarding patient and community safety [27]. Finally, it should not be forgotten that doctors can play a unique, critical role because both patients and their families trust them [23]. The ability of family doctors to communicate with all members of the patient’s family may be especially relevant for preventing traffic injuries in people older than 65 years, since, as Betz et al. [29] pointed out, older drivers with greater family support are more likely to cease driving, if necessary, than those without this support. In this regard, most participants in the present study recognized the importance of family members in several areas, e.g., being the people who first perceive the limitations that may make an older adult unfit to drive and receiving information from the family doctor about an older driver’s potential risks. In addition, our participants recognized the potentially persuasive role of family members’ advice compared to advice from other people regarding the cessation of driving and highlighted the help from family members to travel to their destinations and back as an alternative to driving themselves. Of note, however, is the evidence that when family support is not available, in general the suggestion to cease driving is not considered a useful option.

### Strengths and limitations

This study used a qualitative method to document the opinions of older road users regarding the family doctor’s potential role in their road safety. In this subjective approach to reality, we used an inductive method to analyse the experiences, reflections and thoughts that were made explicit in the participants’ conversation. Our participants’ subjective experiences lend meaning to this study, which aimed to obtain deeper knowledge of the reasons, motivations, beliefs, etc. that underlie older adults’ perceptions and opinions on the role of their family doctor.

We used a single focus group with all 16 participants, due to organizational reasons (the participants attended to a wide range of organized activities at the centre), so we found this unique moment to conduct the focus group. This situation was approached by the moderator, who made an effort to get every participant to speak. The construction of this single focus group was finally beneficial for the study, as it contributed to the identification of emerging issues not contemplated *a priori* and to discursive saturation.

The quality of the relationship between each participant and their family doctor was not addressed in this study, although it should be considered in future studies given its potential influence on the data obtained.

To minimize the lack of representativeness inherent in all qualitative research, a series of profiles was developed *a priori* with segmentation criteria. This approach yielded a sample that we believe was reasonably representative of the most paradigmatic prototypes of the different types of road users over 65 years of age (pedestrians, drivers, passengers, etc.) in our study population.

A further criterion in favour of the representativeness of our sample was that saturation level was reached, as seen from the recurrence of central themes and ideas expressed in the quotations above. In addition, the fact that the transcripts were analysed and coded separately by three authors of this study (with consensus approaching 90 % for most of the categories and their corresponding quotations) is another important criterion in favour of the internal validity achieved through triangulation in the thematic content analysis. As far as we are aware, our work meets the reliability criteria of Lincoln & Guba [32]: credibility, transferability, consistency and neutrality (confirmability).

## Conclusions

In general, older adults are aware that driving at their age poses a risk to their health. Although they trust their family doctors, many of them argue that the issue of driving and health is a matter for other professionals (in whom, paradoxically, they are not very confident), mainly because their physicians have never expressed concerns regarding their driving. However, our results suggest that if family doctors considered road safety during their appointments with older patients, their views would have a considerable impact on these road users, as long as they had access to family support. This opens a window of opportunity for preventive interventions in the primary health care setting. An important aspect that should not be overlooked is the need to address driver safety effectively based on scientific evidence, especially considering the steady aging of the population.

In light of our findings, we suggest some strategies for preventing road accidents in older drivers: (1) developing action protocols for the prevention of traffic accidents in primary care, in order to increase health care users’ trust in their family physicians [28]; (2) considering the implementation of mandatory medical examination and certification for license renewal in older drivers [27]; and (3) providing information on transportation alternatives [33]. These interdisciplinary measures would address a multifaceted problem, since the prevention of traffic accidents affects not only medical care, but also physical therapy, psychology, nursing, public health, social work, and urban planning. In addition, family members and family doctors should work as a team for the benefit of older patients’ health, with due consideration for the possible negative consequences of ceasing to drive.

## Data Availability

The datasets generated and analysed during the current study are not publicly available due to participant confidentiality, but are available from the corresponding author on reasonable request.
